# Proceedings of the World's Temperance Convention, Held in London, August 4th, 1846, and Following Days. With the Papers Laid before the Convention, Letters Read, Statistics, and General Information Presented, &c.

**Published:** 1847-10

**Authors:** 


### Art. XVII.

*Proceedings of the World's Temperance Convention, held in London, August* 4*th,* 1846, *and following Days. With the Papers laid before the Convention, Letters read, Statistics, and General Information presented, &c. &c.—London,* 1846. 8vo, pp. 140.

There are many reasons why we deem it incumbent upon our brethren of the medical profession to take an active part in the investigation which is now being carried on by a large and not unimportant section of the public in this country and elsewhere, with regard to the effects of the habitual use of alcoholic drinks, and the possibility of effectually maintaining the "mens sana in corpore sano" without recourse to them. The fearful array of social and individual evils which may be traced to the *abuse* of fermented liquors, should lead every reflecting mind to consider how far the *use* of them is desirable or necessary; and this inquiry is peculiarly incumbent upon those who assume to themselves the right of guiding the public in all that concerns the welfare of the bodily fabric, whether in health or disease. Their influence for good or evil in this matter can scarcely be too highly estimated. If they are able, after careful consideration of the evidence on each side, to give their sanction to the statements of the advocates of the Total Abstinence cause, that sanction ought not to be withheld; since its weight in the scale of social order and morality demands the open and unqualified expression of it, unrestrained by any fear of ridicule or loss of the world's approval. That they would *knowingly* place their influence in the opposite scale, cannot for a moment be admitted; but there is too much reason to fear that, either from actual ignorance of what the experience of multitudes of all ranks and conditions has now demonstrated, or from a natural tendency to persistence in that sort of *laissez-faire* system which it is so easy to practise and (in this matter especially) so agreeable to their patients, the generality of medical men are at present lending their sanction to a system of most pernicious error. Having long since made up our own minds on this subject, we have determined not to forego this opportunity—the last in our power—of recording our earnest convictions in regard to it; in the hope of leading our readers, if not at once to view the matter in the light in which *we* see it after many years of observation and personal experience, at any rate to inquire and observe for themselves, and to pause before they again recommend or sanction practices which, though comparatively innocent in themselves, aid in perpetuating the direst evils with which our country is infected.

There are, it must be admitted, few cases in which the wish so readily becomes the father to the thought, or in which the feelings are so apt to bias the judgment, as in the consideration of the real utility of fermented liquors. The prevalent ideas of social enjoyment and good fellowship are so intimately associated with the circulation of the bottle or the discussion of a bowl of punch,—most of us have such vivid recollections of the thirst and fatigue of travel alleviated by a glass of good ale at a road-side inn,—

and many a medical man's notions of quiet and legitimate enjoyment after a trying day of professional labour are so closely connected with a comforting tumbler of toddy or brandy-and-water by his fireside,—that it is commonly thought impossible to imagine that the animal spirits can be exuberant without the excitement derived from alcohol, or that the wearied body and mind can derive their needed refreshment from beverages so poor as tea, coffee, or cocoa. When we are regulating the diet of a patient, moreover, how difficult it is to prescribe a rigid abstinence to those who earnestly petition for *only* a glass or two of wine, or a tumbler of beer, per day, as a necessary means of sustaining their fainting strength, or of imparting to them (and this is perhaps the most insidious plea of all) the power of digesting their proper food. And how pleasant it is to preserve the confidence of our patient by thus chiming-in with his humour, rather than by rashly propounding what he may regard as unreasonable crotchets to excite his doubts as to our own sanity. The medical profession in this country, however, is beginning to be awakened from this pleasant *insouciance* by the pressure from without; and to find it necessary to place itself in the midst of the current of human progress, which might otherwise sweep past it and leave its *dicta* among the despised relics of an immoveable conservatism. Some hundreds of medical men of all grades and degrees, in every part of the British empire, from the court physicians and leading metropolitan surgeons who are conversant with the wants of the upper ranks of society, to the humble country practitioner who is familiar with the requirements of the artizan in his workshop and the labourer in the field, have given their sanction (as we shall presently see) to the statement that the maintenance of health is perfectly compatible with entire abstinence from fermented liquors; and that such abstinence, if general, would incalculably promote the improvement of the social condition of mankind. The medical adviser may now shelter himself, therefore, under this high authority; and need no longer be considered a madman, or even an enthusiast, for denying what it has been supposed that the common sense of mankind unmistakeably teaches. The difficulty, however, is to carry this doctrine into practice; and nothing but such a degree of moral courage as can rise superior to temporary ridicule can give success. But our profession is surely one of the last in which that moral courage should be found wanting; for the demands upon it are varied and continual. And in this particular case, it may be remarked, the difficulty is constantly lessening, from the spread of more correct information on the subject; and we have, in fact, known instances in which medical men have *lost* credit with their patients, through urging upon them as necessary those stimulants which their own convictions told them that they were better without.

We need not descant at any length upon the evils of intemperance. The experience of every medical practitioner must have brought its terrible results frequently before his eyes. But whilst thus familiar with its consequences as regards *individuals*, few but those who have expressly inquired into the subject have any idea of the extent of the *social* evils resulting from it, or of the degree in which they press upon every member of the community. On this account we shall preface our inquiry with a few passages from a short paper in the pamphlet whose title we have placed at the head of the present article. This paper, entitled "Intemperance the Great Cause of Crime," consists almost entirely of extracts from recent public addresses of our judges, and from the written statements of magistrates, gaolers, and police-superintendents, whose position furnished them with the means of gaining the fullest information on the subject. The whole of it is pregnant with the deepest and most fearful meaning; and nothing but our limited space prevents us from placing it before our readers in its unabridged condition. We beg their earnest consideration of the following statements.

"Judge Wightman stated in his address to the grand jury at Liverpool, in August 1846, that 'he found from a perusal of the depositions, that one unfailing cause of *four fifths* of these crimes was, as it was in every other calendar, the besetting sin of drunkenness.'

"Judge Alderson, when addressing the grand jury in 1844, at the York assizes, said—'Another thing he would advert to was, that a great proportion of the crimes to be brought forward for their consideration arose from the vice of drunkenness alone; indeed, if they took away from the calendar all those cases with which drunkenness has any connexion, they would make the large calendar a very small one.'

"Judge Erskine declared at the Salisbury assizes in 1844, when sentencing a *gentleman* to six months' hard labour, for a crime committed through strong drink, that ninety-nine cases out of every hundred were from the same cause. Judge Coleridge likewise stated at the Oxford assizes, that he never knew a case brought before him that was not directly or indirectly connected with intoxicating liquors. And Judge Patteson at the Norwich assizes said to the grand jury, 'If it were not for this drinking, you and I would have nothing to do.' One of the judges stated some time ago at the circuit-court in Glasgow, that 'more than eighty criminals had been tried and sentenced to punishment; and that, with scarcely a single exception, the *whole* of the crimes had been committed under the influence of intoxicating liquors. From the evidence that appeared before him as a judge, it seemed that every evil in Glasgow began and ended in whiskey.'" (Proceedings, p. 123.)

So that, according to the testimony of witnesses whose competency and truthfulness no one can call in question, *four fifths* of the entire amount of crimes is the *very least proportion* we can assign to those which are committed under the direct or indirect influence of intoxicating liquors. Let us now call witnesses of another but not less unimpeachable class,—the chaplains of gaols.

"In a late Report of the prisons of Glasgow, an account is given of 3907 individuals, most of whom were committed for crimes respecting which sentence of transportation might be awarded; and, respecting these the Rev. George Scott, chaplain, thus writes: 'Though a number of causes are specified, drunkenness is the most prolific source of most of the crimes in Glasgow. Of the many thousands annually imprisoned, I think it would not be possible to find one hundred sober criminals in any one year. Even the youngest learn this ruinous vice, and when they live by stealing, swallow astonishing quantities of whiskey.' The accuracy of Mr. Scott's observations is corroborated by the new chaplain, in his Report of Glasgow Prisons for 1845. 'To the ruinous habit of drunkenness,' says he, 'may be traced either directly or indirectly the offences of *at least three fourths* of those that come to prison, *females* as well as males. Of this I am convinced, even from their own statements as well as from other circumstances.'" (p. 125.)

The chaplain of the Stirling prison states: "So far as my experience has at present gone, I think that drunkenness is the main cause of crime;" and the Rev. John Clay, the experienced and devoted chaplain of the North Lancashire gaol at Preston, gives similar testimony. "Persons," he says, "who in hard times are led into criminality by destitution, are in better times led into it by drunkenness." To the same effect is the evidence of Mr. J. Smith, governor of the Edinburgh prison. The number of commitments for disorderly conduct arising out of drunkenness, during the year ending June 1844, was 3325; and of those for other offences, the number during the same period was 2385. "I do not hesitate to say," adds Mr. Smith, "that it is my firm belief that, but for drunkenness and the evil and ruinous consequences which follow in its train, there would not have been one fifth part of the number of commitments during the period." The following is Mr. Logan's general summary of similar information obtained from other quarters:

"We collected the following information in July 1844, when visiting prisons in the west and south of Scotland; and the reader will bear in mind that the majority had been committed for theft, and several were about to be removed to our penal colonies. At Greenock, the governor stated that out of 461 prisoners, 297 might be said to have committed their crimes under the influence of drink. At Kilmarnock, Captain Blane believed that he was under the mark, in stating that *four fifths* of the crime there was caused by intoxicating liquors. In Dumfries, the governor was 'warranted in stating that nineteen out of every twenty brought before him were so in consequence of drinking;' and when conversing with *thirty* prisoners out of the total number (forty-two) *twenty-nine* acknowledged that strong drink had been the cause of their imprisonment; and the sitting magistrate stated to the clerk of the police-court, that very morning, that were it not for intemperance, the premises might be shut up for ever. At Ayr, the governor 'had no hesitation in saying that thirty-nine cases out of forty were the fruits of intemperance,' and added, 'if you think proper to visit the prisoners you will find that my statement is pretty correct;' well, we visited each cell, and conversed with every unfortunate inmate; and out of *seventy-three* prisoners there, no less than *seventy* acknowledged that had it not been for these accursed drinking customs, they never would have occupied the lonely cell of a prison. Similar statements were made to us when visiting the prisons of Paisley, Stirling, Hamilton, Dumbarton, Airdrie, and Kircudbright; and what is true of Scotland is to a very great extent the same in England and Ireland....... These facts have all been fully corroborated by the testimony of the respective governors of Millbank Penitentiary and Newgate, London; Wakefield House of Correction; Manchester New Bailey; Newgate and the Female Prison, Dublin; and having visited these prisons and conversed with criminals in each of them (with the exception of Millbank, where it is not allowed), we found that their statements respecting the cause of crime were quite in keeping with those referred to in Scotland." (p. 126.)

We need scarcely, we think, adduce any more evidence in proof of the position that *intemperance is the chief cause of crime*. How fearful then is the responsibility of those who by any means, direct or indirect, encourage this baneful propensity!

To show the dreadful extent to which intemperance prevails, we shall quote from the preceding paper, and from an essay in the same publication, on the Statistics of Temperance and Intemperance, by Mr. T. Beggs, some facts relative to the present condition of the city of Glasgow. On the authority of Sheriff Alison it is stated that in the year 1840 there were in Glasgow, amongst about 30,000 inhabited houses, no fewer than 3010 appropriated to the sale of intoxicating drinks; every tenth house being devoted to the sale of spirits—a proportion unexampled, it is believed, in any other part of the globe. The same gentleman declares that he believes that 30,000 persons, or one tenth of the whole population, go to bed drunk every Saturday night. It appears from an inspection of the registers of the police-station, that not fewer than 25,000 commitments are annually made, on account of drunkenness and disorderly conduct in the streets; and these commitments include no fewer than 10,000 females. A large proportion of the parties so committed are discharged early in the morning and are not brought before the police magistrate, not above a quarter of them being entered upon the records of his office. The annual consumption of ardent spirits in Glasgow is estimated by Sheriff Alison at six gallons per head; making an aggregate of nearly 1,800,000 gallons yearly. The value of this, at the retail price of 15*s.* per gallon is £1,350,000. Now what is the consequence of this as to the health and social condition of the city? "Glasgow," says Dr. Cowan, in his 'Vital Statistics of Glasgow,' "exhibits a frightful state of mortality, unequalled perhaps by any city in Great Britain. The prevalence of fever presents obstacles to the promotion of social improvement among the lower classes, and is productive of an amount of human misery credible to those only who have witnessed it." The returns furnished by Dr. Davidson from the Glasgow Fever Hospital enable us to form some estimate of the influence of intemperance in keeping up this fever, and in aggravating its rate of mortality. Of 249 males admitted during the year ending November 1, 1839, just one half are recorded as "temperate," that is, as never indulging in strong drink to the extent of inebriety; whilst of the remainder, 51 are classed as "a little intemperate," that is, as now and then drinking to intoxication; whilst 73 were "habitually intemperate," drinking ardent spirits whenever they could get them. Of 164 females, 76 or less than half were "temperate," 8 "a little intemperate," and 80 "habitually intemperate." It is evident from these data, that the cases of fever amongst the intemperate part of the working classes must bear a much larger proportion to their number, than among the comparatively sober who constitute (it is to be hoped) the bulk of the community; and that upon the former, therefore, the maintenance and propagation of the disease chiefly depends. The result is still more striking, when the rates of mortality are examined in these three classes respectively. Out of the 201 temperate patients only 28 died, or 1 in 7·2; whilst out of the 212 more or less intemperate, the number that died was 47 or 1 in 4·5. In Dr. Craigie's table of the deaths in 31 cases of fever that occurred in the Edinburgh Royal Infirmary, there were stated to be 15 irregular or dissipated, and only 2 regular; the habits of the remaining 14 are not stated, but they were probably of the "little intemperate" class. This comparison is made, be it remembered, not between drinkers and abstainers, but between different classes of drinkers. The expense in which the city of Glasgow is involved by the fever is estimated at £46,000 per annum; a sum enormous in itself, but like a drop in a bucket compared with that which is squandered in the purchase of ardent spirits.

We shall not pursue this painful inquiry any further; having said enough, we trust, to demonstrate the importance of the subject, to which we would now invite the serious and candid attention of our readers.

A brief historical notice of the origin and progress of the Total Abstinence Movement may not be without its value, in showing how far experience has replied by anticipation to some of the objections which would occur to almost every one who has not given his express attention to the inquiry. It is well known that individuals have risen up from time to time, in all ages, to proclaim the virtues of *aqua pura* as the beverage most conducive to health of body and vigour of mind; and our readers need scarcely be reminded of the cases of Cornaro and Benjamin Franklin, were it not for the remarkable degree in which the strong practical sense of the latter anticipated the conclusions more recently drawn from scientific investigation. "On my entrance into a London printing-house," says Franklin in his 'Autobiography,' "I worked at first as a pressman, conceiving that I had need of bodily exercise to which I had been accustomed in America, where the printers work alternately as compositors and at the press. I drank nothing but water. The other workmen, to the number of about fifty, were great drinkers of beer. I carried occasionally a large form of letters in each hand up and down stairs, while the rest employed both hands to carry one. They were surprised to see, by this and many other examples, that the 'American aquatic,' as they used to call me, was stronger than those who drank porter. My fellow-pressman drank every day a pint with bread and cheese for breakfast, one between breakfast and dinner, one at dinner, one again about six o'clock in the afternoon, and another after he had finished his day's work. This custom appeared to me abominable; but he had need, he said, of all this beer, in order to acquire strength for his work. I endeavoured to convince him that the bodily strength furnished by the beer could only be in proportion to the solid part of the barley dissolved in the water, of which the beer was composed,—that there was a larger quantity of flour in a penny loaf,—and that consequently if he ate this loaf, and drank a pint of water with it, he would derive more strength from it than from a pint of beer." The pious and enthusiastic Wesley, and the ingenious and benevolent Dr. Beddoes, devoted no small amount of labour and reasoning to an attempt to awaken the public mind to the injurious effects of the prevalent use, moderate and immoderate, of fermented liquors; and the latter, amongst other tracts on the subject, published an excellent one in 1808, entitled 'Good Advice for the Husbandman in Harvest,' from which we shall presently make an extract.

Besides these well-known examples, there have always been many who have practised, in a quiet, unostentatious manner, an habitual abstinence from all fermented liquors; and amongst these might be named some who have been remarkable for the amount of mental and bodily exertion which they have been able to sustain. Still it has been the current opinion, sanctioned by the general voice and the usual practice of the medical profession, that the *moderate* employment of fermented liquors of good quality is beneficial, or at any rate innocuous in a great majority of instances; and that where the demands upon the bodily strength are peculiarly constant and severe, efficient aid in meeting them is derived from their use. The cases in which the contrary result has been apparent have been set down as idiosyncrasies, which can afford no rule for general guidance; and those who have ventured to oppose the public *dictum* on this subject have been usually considered at the best as amiable enthusiasts, whose principles, though true as regarded themselves, are not at all adapted for popular practice. But without the guidance of science, and against rather than with the authority of doctors, *the people* have begun to find out for themselves that those well-meaning but impracticable enthusiasts really spoke the truth; and that what has been commonly regarded as universal experience on this matter is nothing better than "a mockery, a delusion, and a snare," having no better foundation than the notion which prevailed until the beginning of the last century, that foul air was beneficial to the sick.

The following is a brief sketch of the origin and progress of the great Temperance Movement.

Association for the purpose of promoting temperance was first conceived and put into execution in the United States of North America, where the inhabitants are far advanced in the knowledge that "union is power," and in the practical skill necessary for applying coalition to a variety of purposes both political and philanthropical. Combinations against drunkenness have existed in America from a very early period. At first they merely insisted on general moderation in the use of strong drinks. Afterwards their rules and regulations became more stringent; and abstinence from ardent spirits at least formed part of the confederate agreement and pledge. About 1815 or 1820, regular societies were formed on this basis in America, and began to extend themselves widely. The method of abstinence from everything intoxicative was at a later period introduced there from Great Britain.

Early in the year 1828 Mr. John Dunlop (a name worthy to be placed by that of Father Mathew, among the benefactors of mankind) began to agitate the subject in Scotland, and took various plans of doing so; such as collecting statistics and proofs of national intemperance; demonstrating the good effects which had followed association against inebriation in America; exposing the evils of the system of compulsory drinking, an usage peculiar to this country; also by travelling about and conversing with influential individuals and philanthropists throughout the country; writing in newspapers, composing and disseminating tracts, lecturing publicly in large towns, &c. Notwithstanding Mr. Dunlop's enthusiastic and energetic proceedings, he did not succeed in gaining any regular disciples to the cause till about the latter end of 1829 and beginning of 1830, at which period the Greenock, Glasgow, Edinburgh, Dundee, Paisley, and other societies were instituted in Scotland; the Bradford Society in Yorkshire, by Mr. Forbes; and the Old British and Foreign Temperance Society in London, by Mr. Collins of Glasgow. Mr. Dunlop had proposed that all wines should be abandoned as well as spirits, but only partially succeeded in that point.

In the summer of 1829 Dr. Edgar of Belfast, without knowledge of Mr. Dunlop's proceedings, set on foot the Irish temperance movement, assisted by Mr. George Carr; and it is believed that the first European temperance society was established by the latter at New Ross about June or July 1829; Mr. Dunlop's first societies not having been established till October of that year. Thus the Irish and British movements were separate and independent in their commencements.

Things continued on the original footing for some years, with only in general a pledge against the use of ardent spirits. But it soon became evident to reflecting persons engaged in the cause, that half measures would not suit the urgency of the case, and that a prodigious and nearly universal national evil must be met with correspondent strength of remedy. It was remarked, too, that all drunkards who were really reformed by joining the societies, not merely conformed to the actual rules, but uniformly abstained from using anything intoxicative. Many individuals connected with the societies accordingly made it their practice to abstain totally, and in 1832 it is believed that the Paisley institution made total abstinence a part of their regulations.

Teetotalism, or total abstinence from all intoxicating drinks, was not, however, fairly established generally till 1834; when the seven men of Preston, assisted by Mr. Pollard of Manchester, and others, started societies fairly and exclusively on the teetotal principle. The published lectures of Mr. Livesey of Preston constitute an era on this subject; and the labours and lectures of a gentleman in the iron trade at Liverpool (whose name we forget at the moment) were extremely useful at this period.

Shortly after this the New British and Foreign Temperance Society was established at London on teetotal principles, under the management of Messrs. Janson, Oxley, Meredith, Green, and others; and very soon almost all the original temperance societies in Great Britain adopted the rule of total abstinence. The doctrine was pushed over into Ireland by the Liverpool gentleman we have mentioned, to be brought into the most brilliant success in due time afterwards by Father Mathew.

About 1836 the teetotal element was established in the American temperance societies, which, it is believed, are now universally conducted on this principle.

The total abstinence reform has extended itself successfully into Canada, New Brunswick, India, the West Indies, and our other colonies. It has also made its way into the South Sea Islands, and elsewhere among the half-cultivated races; and has been partially adopted in Sweden and other parts of the North of Europe.

The following are the terms of the certificate to which we have referred, as having received such numerous signatures of medical practitioners, including those of many of our most distinguished physicians and surgeons:

"We the undersigned are of opinion—

"1. That a very large proportion of human misery, including poverty, disease, and crime, is induced by the use of alcoholic or fermented liquors as beverages.

"2. That the most perfect health is compatible with total abstinence from all such intoxicating beverages, whether in the form of ardent spirits, or as wine, beer, ale, porter, cider, &c. &c.

"3. That persons accustomed to such drinks may, with perfect safety, discontinue them entirely, either at once, or gradually after a short time.

"4. That total and universal abstinence from alcoholic liquors and intoxicating beverages of all sorts would greatly contribute to the health, the prosperity, the morality, and the happiness of the human race."

Let us say, however, *in limine*, that whilst taking upon ourselves the earnest advocacy of these doctrines, we by no means wish to identify ourselves with all that has been written and uttered by the disciples of the Total Abstinence system. Too often their intemperance has passed from their cups to their language; the finger of pharisaical scorn has been pointed at the "moderate drinkers," whose consciences have not yet told them that there is any harm in the temperate use of fermented liquors; and even those who agree with them in their leading principles, and who join with them in their practice, but who hesitate at sanctioning all that ignorant enthusiasts think fit to assert, have been stigmatised as enemies rather than as friends to the great cause of emancipation. Now we most fully recognise the importance of earnest and awakening appeals to those who are sunk in the lethargic slavery of one of the most brutalising of all sensual indulgences; but we are certain that exaggeration never ultimately serves the interests of truth. No words *can* depict too strongly the evils of intemperance. No appeals *can* be too urgent or awakening to the blunted feelings of those who are ruining themselves both for time and eternity by an habitual indulgence in this overpowering propensity; but surely there is plenty of matter for the advocates of abstinence, without going out of their way to condemn those who maintain that fermented liquors are the gifts of God, to be *used* in moderation but not *abused.* We are quite sure that the manner in which their public proceedings have been conducted has kept many aloof, who would have been most valuable and influential advocates of this great cause of social and individual reformation. The fact we believe to be, that a large proportion of the intemperate denunciations and rash statements to which we allude, have been put forth by men who have themselves felt all the tyranny of this dreadful slavery; and (as we have been informed by some most competent observers) they feel, on their emancipation from it, a sort of excitement that is almost uncontrollable, urging them to bear public testimony to the evils from which they have escaped, and infusing into that testimony a strength that makes it operate powerfully on the minds of those whom they desire to awaken, whilst it leads them (with the want of discrimination natural to men of imperfect education) to express the most unmitigated reprobation of those more especially who profess themselves friends of temperance, but who do not feel called upon to preach or to practise total abstinence.

Now we are quite content to brave their condemnation for the sake of what we consider to be truth; and feeling satisfied, as we just now said, that the interests of truth cannot be served by exaggeration, we think it right fearlessly to state that we cannot, with them, affirm that we consider alcohol in all its forms to be nothing else than a poison. We cannot conscientiously go the length of denying that under any circumstances, whether of health or disease, the administration of alcohol can be justified. We believe that if the whole world could be *really temperate* in the use of fermented liquors, there would be no need of Total Abstinence societies. But we advocate their principles, because sad experience has shown that a large proportion of mankind *cannot* be temperate in the use of fermented liquors, and that nothing short of total abstinence can prevent the continuance, in the rising generation, of the terrible evils which we have at present to deplore;—because experience has further shown than the reformation of those who are already habitually intemperate cannot be accomplished by any means short of entire abstinence from fermented liquors;—and because experience has also proved that this reformation cannot be carried to its required extent without the moral influence of the educated classes. Such influence can only be afforded by *example.* There is no case in which its superiority over mere precept is more decided and obvious than in this. "I practise total abstinence myself," is worth a thousand exhortations; and the miserable failure of all the advocates who cannot employ this argument should lead all those whose position calls upon them to exert their influence (and who are there who do not possess *some* means of thus doing good?) to a serious consideration of the claims which their duty to society should set up in opposition to their individual feelings of taste or comfort.

Without setting ourselves up as apologists for the managers of Total Abstinence Societies, we may remark that this, like other great movements, has been mainly brought about by the agency of individuals, whose very enthusiasm necessarily renders them somewhat one-sided in their views. It is for the cool-judging philosopher to place these views in their true light, and thus to guide mankind in forming a just appreciation of them; but such a movement might be retarded for centuries, or might never take place at all, if there were no one-sided enthusiasts in the world, and it were left to the philosophers to set it going. Now the leaders of the Total Abstinence movement have conscientiously felt that a charge has been laid, as it were, upon their shoulders, to rid the country of intemperance; and they can fairly plead the enormity of the disease, and the difficulty of completely eradicating it, as an apology for the severity of their cure. There are so many apertures, they affirm (and with justice), by which men contrive to escape from the Abstinence principle,—creeping out, like cunning foxes, in search of the object of their craving,—that every hole must be stopped up, every apology for a recourse to alcoholic liquors cut off. We admit the danger, and the necessity for the utmost caution in the avoidance of it. But still we do not think that those of the professed advocates of Total Abstinence, who deny the possibility of *any* benefit from the use of alcohol, have taken up a defensible ground; and the argument for the stringency of the pledge should rather be based, in our estimation, upon the risk of abuse, which the slightest violation of it has been found by experience to involve, than upon those asserted "poisonous" properties, which it assuredly does not possess in a degree nearly so strong as many of our most valued medicines. Dissenting as we thus do from much that has been uttered from the Teetotal press and platform during the last ten or twelve years, we yet must in justice admit, that when so large a number of parties are concerned (and these chiefly of very limited education), as in the present case, it is somewhat unreasonable to expect perfect wisdom in every mouth, or that zeal in so good a cause should not sometimes get the better of discretion. And the leaders of the movement may fearlessly ask, what assocation of such a size,—political, ecclesiastical, or philanthropic,—could bear to be tried by a severe test on this point?

In the exercise of our own duty as cool-judging critics, we now propose to inquire in the first place into the present state of our knowledge as to the physiological action of alcohol on the human body; next, to consider how far the results of the comparative experience of those who make habitual but moderate use of fermented liquors, and of those who entirely abstain from them, under a variety of circumstances, warrants the assertion that total abstinence is invariably (or nearly so) compatible with perfect health, or is even more favorable to health than habitual but moderate indulgence; and finally, to endeavour to deduce from these data such conclusions with regard to the therapeutic use of alcohol, as may cause its employment by medical men to be attended with the greatest possible amount of good and the least admixture of evil.

Our knowledge of the physiological action of alcohol, though far from being sufficiently complete to afford a specific determination of its hygienic or therapeutic value, is yet quite sufficient to guide us in the inquiry; and we shall accordingly state briefly the points which may be regarded as in our apprehension most satisfactorily made out. We believe that no physiologist of repute would now be found to maintain any other doctrine in regard to the materials of the albuminous tissues of the animal body, than that propounded a few years since by Mulder and Liebig; namely, that they are derived exclusively from those alimentary substances whose constitution is similar to their own; so that the non-azotized compounds cannot enter into the composition of more than a very small part of the animal fabric. This doctrine, when first put forth, was received with a degree of hesitation and distrust proportioned to its novel and startling character; but the testimony in its favour has been gradually though quietly accumulating, so that it now commands very general if not universal assent. By the term "albuminous," we mean to designate all those tissues which can be formed at the expense of albuminous matter; and this category includes the gelatinous and horny tissues, as well as those which possess a composition more nearly allied to that of albumen; for we know that the former, as well as the latter, must be generated from albumen during the incubation of the egg, as well as during after-life, when neither gelatine nor horny matter exists in the food. The only tissues in the animal body of which albumen does *not* form the principal basis, are the adipose and the nervous. In *both* these it is probable that the membranous *walls* of the cells and tubes have (like similar membrane elsewhere,) an albuminous composition; the *contents* of these cells and tubes being of a non-azotized character in the latter case as in the former. For it has been pointed out by Valentin (Lehrbuch der Physiologie, Band I, p. 174) that although the substance of the brain and nerves appears to yield an azotized fatty acid when analysed *en masse*, the supposed composition of this acid (which is quite an exception to all chemical probability) may be accounted for by regarding it as a *mixture* of albuminous matter and ordinary fat, which is exactly what might be anticipated on anatomical grounds.

All our present physiological knowledge, then, leads to the decided conclusion that alcohol cannot become the pabulum for the renovation of the *muscular* substance, which process can only be effected by the assimilation of albuminous materials in the food; and that the habitual use of alcohol, therefore, cannot add anything to the muscular vigour. And this conclusion receives most striking confirmation from the well-known fact, that, in the preparation of the body for feats of strength, the most experienced *trainers* either forbid the use of fermented liquors altogether, or allow but a very small quantity to be taken; their trust being placed in a highly nutritious diet, active muscular exertion, and the occasional use of purgatives which purify the blood of the products of decomposition or draw off superfluous alimentary materials.

That alcohol has some peculiar relation to *nervous* matter, would appear from its power of stimulating the nervous system to increased action; but this power, although coincident with a certain relation in their chemical composition, could not be predicated from the latter, since ordinary fat, which has no such stimulant effect, has a closer chemical relation to nervous substance than is possessed by alcohol. Whether alcohol is capable, by any transformation, of being converted into nervous matter, is a question which we have at present no data to determine; but there can be no doubt that this tissue may be formed equally well from other ingredients of food, which have not like it a stimulant effect. It cannot, therefore, be a *necessary* pabulum to the nervous system; and its peculiar virtues as an habitual article of diet, if such there be, must be looked for in its stimulating qualities.

But, it may be maintained, although alcohol is not requisite or useful as a pabulum for the tissues, it is most efficient as a combustible material, serving to keep up the heat of the body in extreme cold, and to defend it against the effects of vicissitudes of temperature,—in common language, "to keep the cold out." Now this at first sight appears a very cogent argument for its use under certain circumstances, if not for its regular employment; but when its effects are more closely examined, it will be found that neither physiological science nor the results of experience sanction such a proceeding. The maintenance of the animal heat chiefly depends, as all our readers must be aware, upon the formation of carbonic acid and water by the oxygenation of hydro-carbon contained (probably in various forms) in the blood. Now the ingestion of alcohol, so far from promoting, *checks* the oxygenating process; as was shown long since by the result of the experiments of Dr. Prout, who invariably found the quantity of exhaled carbonic acid to exhibit a marked decrease after the ingestion of alcoholic drinks, other circumstances remaining the same. Subsequent experimenters upon the respiratory process have met with the same results; and they are confirmed by the fact ascertained by Bouchardat, that when alcohol is introduced into the system in excess, the blood in the arteries presents the aspect of venous blood, showing that it has not undergone the proper oxygenating process. Now although we may not understand the reason of this (although it seems to be referable to the well-known power of alcohol to prevent or retard chemical changes in organic substances), the fact is of the utmost importance.

The inference to which we are thus conducted by physiological reasoning, instead of being negatived by general experience (as it is commonly supposed to be), is fully confirmed by it. The Esquimaux, Greenlanders, and other inhabitants of the coldest regions of the globe, effectually maintain their animal heat by the large consumption of fatty matter; and whatever may be the *temporary* effect of an alcoholic draught, we believe that all arctic and antarctic voyagers agree that *continued* resistance to cold is most effectually maintained without alcohol, or at any rate with a much smaller quantity of it than is commonly thought necessary. A very striking proof of this is afforded by the arrangements recently made for the overland arctic expedition, on which the best authorities have of course been consulted by Government. In the programme of these arrangements it is expressly stated, that *no fermented liquors* are to be used by the parties who proceed upon it. We have heard many of the now almost extinct race of stage-coachmen, who had been induced to give up their former habit of imbibing a glass of ale or of brandy-and-water at every stage, and to substitute an occasional cup of hot coffee and a rasher of toasted bacon, speak most decidedly in favour of the superior efficacy of the latter system; and we doubt if any man who had the resolution to adopt it, ever returned to his habits except from the love of liquor. We are strongly inclined to the belief that much of the reputed warming effect of alcohol is due to the hot liquid with which it is usually combined when used for that purpose. A tumbler of hot brandy-and-water, of whiskey-toddy, or negus, is doubtless a very comfortable beverage when imbibed on a cold night on the top of a coach; but our own experience, and that of many others who have tried the experiment, warrants the belief that a cup of hot tea, coffee, or cocoa will have quite as much warming influence, whilst a *cold* alcoholic drink will be nearly if not quite ineffectual. The only cases in which we conceive that alcohol in any form can be more useful than other compounds of hydro-carbon as a heat-producing substance, are those in which all the combustible material of the body has been used up during the progress of a fever or other exhausting disease, and in which the state of the digestive system prevents the reception of any other kind of pabulum into the circulating current. To these we shall hereafter more particularly refer.

Before leaving the question of the heat-producing powers of alcohol, we should advert to an explanation which has been offered, of the diminution in the amount of carbonic acid exhaled after its use; since this explanation, if correct, might vitiate the theoretical part of our argument, though it could not affect the results of experience. It has been stated by no less an authority than Liebig, that this diminution of carbonic acid is owing to the comparatively small proportion of *carbon* in alcohol; the heating power of that substance being chiefly due to the *hydrogen* it contains, which is exhaled from the lungs in the form of the vapour of water, so that, whilst the alcohol is being carried off from the blood, the proportion of carbonic acid to watery vapour, in the products of the combustive process, will be unduly low. This may possibly be true; but it is not the whole truth. There are other substances, as Dr. Prout has shown, whose ingestion is followed by similar results, to which no such explanation is applicable; of this nature is strong tea, especially green tea. And moreover, the experience of Dr. Prout would lead to the decided conclusion, that the presence of alcohol in the blood prevents the extrication of matters whose retention is injurious to it, and for whose removal the respiratory process is the appropriate means. For whilst the diminution in the amount of carbonic acid exhaled continues as long as the effects of the alcohol are perceptible to the individual who has swallowed it, these effects no sooner pass off (which they did in Dr.

Prout's individual case, with frequent yawnings and a sensation as if he had just awoke from sleep), than the amount of carbonic acid exhaled *rises much above* the natural standard,—thus giving, it would seem, unequivocal evidence of the previous abnormal retention of carbon in the system, of which it is only able to free itself after the alcohol has been burned off. And this view is further confirmed by the fact which experience has forced upon men who are so far most unwilling adherents to the abstinent system,—that alcoholic liquors ingested *during* the performance of severe labour, in very hot situations, cause a very rapid and decided failure of the strength; so that men who drink largely of such liquors in the intervals of their work, are obliged to abstain from them whilst their labour is in progress. The physiologist well knows that the quantity of hydro-carbon carried off by the lungs diminishes as the external temperature rises; and one of the reasons for the oppressive influence of a continued exposure to great heat is probably to be found in the obstruction which it presents to the extrication of that amount of carbonic acid, whose removal is necessary for the depuration of the blood. Now, if alcoholic liquors be ingested in this state of the system, and interpose (as we have endeavoured to show that they do) a still further obstacle to this process, the result would be precisely what experience demonstrates,—namely, the flagging of the powers of the system, from the imperfect purification of the blood.—Thus, put the subject in what light we may, theory and practice here go hand in hand in guiding us to the conclusion that alcohol is not more efficacious than other pabula for the combustive process, except in certain disordered states of the system, to which we shall hereafter refer; and that its habitual use cannot be defended on the ground of the necessity for supporting the heat of the body by its means during exposure to very severe cold, whilst it is positively injurious when the surrounding temperature is high. We shall presently adduce other evidence upon this latter point, from the experience of those who have resided in tropical climates.

It appears, then, that the physiological influence of alcohol upon the system, under all ordinary circumstances, cannot be attributed to anything else than its *stimulant* character; and it is almost a self-evident corollary from this proposition, that its *habitual* use even in moderate quantities can exert no beneficial effects. For the healthy fabric should be quite capable of maintaining itself in vigour upon a proper diet and with a due quantum of sleep, exercise, &c., without any adventitious assistance; and if it be not, assistance should be sought from alterations in diet or regimen, or from remedies which tend to promote the regular play of its functions, rather than from stimulants, which may produce in some of these a temporary excitement, but which thus tend to destroy the balance of the whole. The very nature of a stimulant is to produce a subsequent depression, and to lose its force by frequent repetition. The depression is proportional to the temporary excitement; and the loss is thus at least equivalent to the gain. And when a stimulus loses its effect as such by frequent repetition, it is still felt as being necessary to bring the system up to par, an increased dose being required to elevate it higher. Thus, as is well known, those who habitually employ fermented liquors for the sake of their *stimulating* effects, are led on from small beginnings to most fearful endings; and the habit, growing by what it feeds on, becomes a necessity. No pretext is more commonly given out as an apology for the habitual use of fermented liquors, than the aid which a moderate employment of them is thought to afford to the digestive process. But we maintain that, where a man duly observes the laws of health, the appetite will always desire the amount of food which the system needs, and the stomach will be able to digest it. If health is to be measured by the capacity for eating, then the habitual moderate use of fermented liquors may be conducive to it; but if the increase in this capacity which they produce be of no service to the economy at large, they cannot have any other than an injurious effect, by leading us to overtask the powers of our digestive apparatus. Thus, as Liebig has very well pointed out, the residents in warm climates who take stimulants before their meals, in order to make up for the deficiency of appetite, act upon a most unphysiological and ultimately injurious system; forgetting or being ignorant that the real demand for food is much less when the surrounding temperature is high, and that the diminished appetite really indicates the diminished wants of the system. In a large proportion of the cases in which the habitual employment of fermented liquors has really a show of utility, we are quite certain that a copious use of cold water externally, and the substitution of it for more stimulating beverages, will be found in the end to be the most wholesome practice, tending (as large experience has shown that it does) both to improve the appetite and to invigorate the digestive powers.

We do not go so far as to maintain that *no* exceptions are to be made to this rule; but we are satisfied that these exceptions are much fewer than is commonly supposed; and that they are to be made rather in cases where some temporary disturbing cause is acting upon the system, than in those in which there *seems* to be an habitual want of assistance.

In like manner, we believe that the nervous system can derive no benefit from the habitual use of fermented liquors; since, in a healthy state of body, it ought to be equal to the work it is called upon to perform; and, if over-tasked, it must be renovated by repose. Doubtless it may be stimulated to increased temporary activity by the use of alcohol; but this activity can never be long sustained; and in the state of subsequent depression, the body is more than usually liable to the influence of morbific causes. There is no part of our frame which requires nicer management, or which is more rapidly acted upon by influences from without or from within, than the nervous system. The regular employment of it, if well directed, and carefully supported by attention to everything that promotes the general health, may be carried to a marvellous extent; and yet, in some peculiarly susceptible constitutions, the least indisposition gives rise to a feeling of nervous depression, which might seem to demand the use of stimulants for its removal. In the majority of cases, however, this feeling of depression is the result of habitual inattention to the laws of health; and, although it may be temporarily removed by alcohol, yet the evil is only palliated for a time; and the very means employed lays the foundation for a future increase of the feeling of depression, requiring an increase of the stimulus for its removal. We may appeal to universal experience in support of our doubts, whether those who have had frequent recourse to alcoholic stimulants for the removal of nervous depression, arising from previous exhaustion by over-work, or from disorder of some other function of the body, have been able to stop at any one point; or whether they have not, to produce the same effect upon their feelings, been obliged to increase the dose, the more frequently it has been repeated. This we conceive to be the great and palpable distinction between the effects of a stimulant which excites, and a pabulum which supports, the system. The former needs to be increased in proportion to the frequency with which it is employed. The demand for the latter varies merely in accordance with the amount of renovation to be effected.

But it is often asserted, that although stimulants may be dispensed with in temperate climates, the habitual use of them is necessary to aid the system in resisting the enervating influence of extreme heat. Let us see how far the results of experience, when carefully sifted, bear out this doctrine. In the first place, we presume, it will be readily admitted by our readers, that the effects of *excess* in the use of fermented liquors are far more injurious in hot than in temperate climates. A very considerable proportion of the mortality in the stations which have the reputation of being the most unhealthy, is so directly traceable to such excess, that its continuance can scarcely be accounted for, except on the principle of "a short life and a merry one." Some years since, whilst ourselves stationed in the West Indies, we conversed with a gentleman resident in Tobago, who informed us that the average annual mortality amongst the Europeans of that island was *one in three.* Upon inquiry into the habits of the residents, we found that intemperance prevailed amongst them to a most fearful extent; few getting up in the morning without their glass of sangaree (wine and water), and the strength of their beverage being gradually increased during the day, until it arrived at neat brandy at night. He further spoke of it as no uncommon occurrence for a party of friends who had met at a drinking bout, to be summoned within two or three days to the funeral of one or two of their number. Our informant was himself apparently quite indisposed to recognise between these occurrences any relation of cause and effect; and was obviously under the belief, that if it were not for the protecting influence of good wine and brandy, his life would be worth a yet shorter purchase. Our readers will probably form a different conclusion. We have on various occasions sought for information from those who had best preserved their health during a long residence in tropical climates, as to their habits in regard to the use of alcoholic liquors, and have almost invariably found that they had practised extreme moderation, if not total abstinence. All medical men who have practised in India agree in attributing the large proportion of cases of severe disease which present themselves among Europeans in that country to the immoderate indulgence in fermented liquors. A statistical proof of it is afforded by the fact quoted by us in a former Number of this Journal (January, 1841), in regard to the experience of the British army in Bengal, in which temperance societies (on the old plan of abstinence from distilled spirits only) had been established a few years previously. We must refer to our former abstract for a fuller statement of the results of the disuse of ardent spirits and the diminished consumption of other fermented liquors; and shall only here state that the returns drawn up by the Inspector-General for the first six months of 1838 show that the average daily per centage of sick belonging to the Temperance Society (about one third of the whole strength) was only $3\frac{2}{3}$, whilst the daily per centage amongst the remainder was $10\frac{1}{5}$. Even this result does not give the most favorable view of the case; for many men joined the Temperance Society whose constitutions had been ruined by previous dissipation, and several such were habitual tenants of the hospital until invalided. Since that time, the Total Abstinence principle has been introduced among Europeans in India and other tropical countries; and, we are assured, with the most favorable results. There has been no want of satisfactory medical testimony in its favour. Indeed, all our best writers on tropical diseases are most explicit on this point. And we may here give the evidence recently given by Mr. Gardner, now superintendent of the Botanic Gardens in Ceylon, a well-educated surgeon, who spent several years of most active exertion in Brazil, and who penetrated into that country further than any other scientific European. During three years' travelling in that climate, under constant fatigue and exposure to vicissitudes of weather and irregularity of living, his only beverage, besides water, was tea, of which he had laid in a large stock previously to his departure from Pernambuco. He was told when he arrived at Brazil, that he would find it necessary to mix either wine or brandy with the water which he drank; but a very short experience told him, not only that they are unnecessary, but that they are decidedly hurtful to those whose occupations lead them much into the sun. "Whoever drinks stimulating liquors," he says, "and travels day after day in the sun, will certainly suffer from headache; and in countries where miasmata prevail, he will be far more likely to be attacked by the diseases which are there endemic."

Now this testimony, from those who *have tried* the experiment of total abstinence in tropical climates, and who have watched its results in others, must surely be regarded as of greater weight than any vague notion to the contrary, however prevalent such notion may be; more especially as it corresponds exactly with what might be predicated upon scientific grounds. For, as we have already shown, the introduction of alcohol into the blood obstructs its depuration by the respiratory process; more especially when the surrounding temperature is high, and the natural exhalation of carbonic acid is consequently diminished. Hence the system is subjected to the injurious influences of an imperfectly decarbonized and aerated blood; and the liver is called upon to do what the lungs are prevented from effecting, —the foundation being thus laid, in the habitual stimulation of the liver to undue functional activity, of inflammatory disease in that organ.

The testimony of those who are exposed to *vicissitudes* of climate is perhaps even more valuable than that of those who have to sustain continued heat or severe cold; and under this aspect we regard the evidence of intelligent seamen as of peculiar importance, in addition to the force it derives from the well-known attachment of their class to spirituous liquors. That such regard the total abstinence principle as at any rate a *safe* one, may be inferred from the circumstance that it is now carried into practice in a very considerable part of the merchant service in this country, and in a still larger proportion of American vessels; and that the adoption of this plan is not known to occasion any difficulty in obtaining crews for the "temperance ships," when a fair compensation is made in the superior quality of the provisions and allowances, or in the rate of wages, as an equivalent for the "stoppage of the grog;" in fact, such ships are often in positive request. And it is not a little worthy of note, that lower rates of insurance are frequently taken upon "temperance ships," than upon those in which the usual allowance of spirits is continued; it being well known that a large proportion of losses at sea are due to the intemperance of officers and men. We consider that an immense improvement was made in the victualling of the navy, when the allowance of grog was diminished, and coffee, cocoa, &c. were substituted; and we trust that the day is not far distant when the total abstinence principle may be recognised as worthy of government support in the army and navy, instead of being (as at present) checked or discouraged by the strong temptations to indulgence which are placed so completely in the way of the men, as to require great moral courage on their parts to resist them habitually. That the moral condition of sailors is more likely to be raised by the universal extension of the abstinence system amongst them, than by any other single measure of improvement, is unhesitatingly declared by all who have had experience of the superior conduct of the sailors on board the "temperance ships;" and we feel assured that the "cat" may be discarded when the grog is thrown overboard; at least two thirds of the offences now punished by flogging having their origin directly or indirectly in alcoholic excitement.

That there are peculiar difficulties attending the complete withdrawal of the allowance of spirits in the naval service, we freely admit; and it is well that these difficulties should be openly stated, in order that they may be fairly met, and, so far as possible, counteracted. We have requested a distinguished medical officer attached to the late Antarctic expedition, to place us in possession of his opinions on this point; and we are sure that those who are acquainted with the scientific reputation of Dr. Joseph Dalton Hooker will consider the declared results of his experience under such trying circumstances as highly important. In reply to our question, whether the habitual use of fermented liquors may be *safely* dispensed with on board-ship, he thus writes:

"I should say clearly so, and with benefit, too: *provided the water be good.* For the comfort of the men the water should be palatable; and this is far from being universally practicable. The *officer* can vary his viands and drink so much, that to have no spirits is no loss to him; but there is no substitute for grog to the sailor. Beer is too bulky; lemonade soon palls when daily used, and would not agree with all. Cold tea is not palatable to every one, even if recommendable; and the sailor gets hot tea once a day as it is, which in hot weather is almost once too often. You must not judge of the navy by the merchant service. In the latter, the sailor joins for immediate profit, and is willing to go through the voyage with bad water and no spirits, for it only lasts a few months or years; and, in joining an abstinence ship, he does not forswear grog for ever, and has opportunities of varying his beverage with his ship. The good navy sailor, on the other hand, ships for forty years (at least such are the men we want and prize); and for five, or even seven years' commission at a stretch, in a very hot climate, where the water is bad, perhaps, he has but one diet, and no prospect of its being altered.

"Perhaps the gravest objection to abolishing fermented liquors in the navy allowance is, that you cannot do so with the officers. They are allowed to buy and lay-in private and mess-stock, and the service allows them stowage. It is not so with the sailor. He is prohibited from laying in a sea-stock, both because no room is allowed him to store it, and because he could not afford it, or be trusted if he could. In the army, where there is little or no communication between officers and men, and no intimacy, this would not tell so heavily as on board-ship, where every one has a great fellow-feeling with his shipmate, and where partiality in the treatment of any class, with regard to the withholding of what each in his station is accustomed to on shore, could not fail to produce a very strong feeling. These are, however, secondary considerations. *Allowing the water to be palatable,* I have no hesitation in saying that the habitual use of the spirit may be *beneficially dispensed with,* as far as the health of the crew is concerned."

Two points in the foregoing extract are particularly worthy of note: first, the importance assigned to the goodness of the water; and secondly, the stress laid upon the example of the officers. It is well known that the substitution of iron tanks for wooden casks has been of the greatest benefit in improving the quality of the water on board-ship, as well as in saving stowage-room; but much still remains to be done. If the accounts which we have recently heard, of the success of the application of electricity to the decomposition of those minute quantities of organic matter in water that has been long kept, to which its taint is due,—an application which has been recently made by the well-known electrician Mr. Andrew Crosse, and for which he has taken steps to secure a patent—should prove correct, a great boon will have been conferred on our naval service, which will render it much easier, we hope, to extend to it the still greater boon of the total abstinence reformation. With regard to the influence of the example of the higher classes, we see that it is far stronger on board-ship than on shore, either for evil or for good. Let the officer once determine to forego his moderate allowance of wine, spirits, or malt liquor, and the seaman will be easily induced to follow his example. The medical officers of the navy have it in their power to set on foot a reformation, the glory of which shall far surpass that of the greatest victories which history records; for let *them* begin and persevere, without fear of ridicule or obloquy, and we feel assured that they will make certain progress, though it may be slow.

In reply to our second query, whether the abstinence in cold climates is attended with positive benefit, Dr. Hooker writes:

"I do think that the use of spirits in cold weather is generally prejudicial. I speak from my own experience. *It is very pleasant.* The glass of grog warms the mouth, the throat, and the abdomen; and this, when one is wet and cold, with no fire, and just before turning into damp blankets, is very enticing; but it never did me one atom of good: the extremities are not warmed by it; and when a continuance of exertion or endurance is called for, the spirit does harm, for then you are colder or more fatigued a quarter or half an hour after it than you would have been without it. Several of the men on board our ship, and amongst them some of the best, never touched grog during one or more of the antarctic cruises. They were not one whit the worse for their abstinence, but enjoyed the same perfect health that all the crew did throughout the four years' voyage. Many of our men laid in large stocks of coffee, and when practicable had it made for them after the watch on deck. These men, I believe, would willingly have given up their spirits in exchange for coffee; but we could not ensure them the latter on the requisite occasions. To the southward of the antarctic circle, or of lat. 50°, you may say, it blew a gale three days out of five; there was always a heavy swell running; the whole ship and bedding were damp from condensation, where not so from shipping seas; the atmosphere of the lower deck (with hatches battened down) such that you could not see from one mess-table to another; and this for days together. There is neither standing, sitting, nor lying in comfort. All hands, officers and men, up and ready; the one watch on deck, the two others on the *qui vive* for any emergency. In cruising amongst the ice, the ship is perhaps put about every half-hour; and we have been for sixteen hours in this state. Every time we go on deck we are drenched with cold salt water, which sometimes freezes as it falls; and when you go below, there is really nothing to do but 'lick your paws,' as the men say. Nothing hot can be got."

Certainly a more uncomfortable situation, short of positive danger, can scarcely be imagined. Let us see what Dr. Hooker says of the use of spirits on these occasions, in answer to our third query—whether there exist *any* circumstances, which in his opinion render the *occasional* use of alcoholic liquors beneficial.

"This is perhaps as extreme an instance as I could bring forward of the demand for spirits. Now I do not believe that to 'splice the main-brace' half a dozen times, or even more, in this sixteen hours, would do any good in the way of giving strength; but to refuse the men some grog would be a great hardship. I have seen grog given, half a gill at a time, thrice (I think) under such circumstances, with no perceptible harm; but I do not suppose it did any good; and more would, I am sure, have done mischief. The fact of giving it did good in one way;—it made the men joyful, not from excitation, but as we all rejoice on cutting the Christmas pudding; and I quite believe that under that contiuued exertion the bad effects were dissipated. But this is a very different thing from doing any real physical good. I can well suppose the effect to have been, though inappreciably, the contrary. Of one thing I am sure, and that is, that no one was more ready for a repetition of the exertion from taking the stimulus; the intervening time was more pleasantly and comfortably passed. It may be a question whether, granting the spirits to have done some good towards exhilarating, when no modern appliances could be available, it would be desirable to withdraw it on such occasions. It is a choice of evils perhaps.

"I know of only one occasion on which the spirits appeared indispensable; and that was, when a little more exertion at the crowning of a mighty and long continued effort was demanded. Thus the ship, when sailing in the pack-ice, is sometimes beset, or falls to leeward into the lee-ice. This takes two or three minutes; but if there is much wind, it takes many hours to get her out. Not being in command, the sails are of no use; and the ice prevents her moving in any way but with it to leeward. Under these circumstances, the only way to get her out is by fastening ropes from the ship to the larger masses of ice, and warping her out by main force against the wind. Now I have seen every officer and man in the ship straining at the capstan for hours together, through snow and sleet, with the perspiration running down our faces and bodies like water. Towards the end of such a struggle, at the mighty crowning effort, I have seen a little grog work wonders. I could not have drunk hot coffee without stopping to cool; nor if I had, do I think it would have supplied the temporary amount of strength which was called for *on the spot* under circumstances like this.—These, however, are extreme cases, which do not affect the sailor in his ordinary condition, and which any ship might be well prepared for."

Fully agreeing with Dr. Hooker, that we know of nothing which, under such trying circumstances, could be advantageously substituted for the alcholic stimulus, we may add the remark that, where the habitual use of it is relinquished, a much smaller amount of it will suffice to produce the required stimulation, than when a large allowance is daily imbibed. Every medical practitioner must be aware of the necessity of regulating the quantity he administers for any particular object by the usual habits of his patient; a single glass of wine doing that with one, which an entire bottle would scarcely effect with another more seasoned vessel. We must not omit Dr. Hooker's conclusion.

"The great practical difficulty on board-ship is, that you have no available substitute for bad water but good grog, as the sailor is at present situated. I cannot, however, but think that, with more attention to the comforts of the sailor, his own love of liquor would diminish; and that he might be weaned from it by the officers, though the depriving him of it by the Government would be a dangerous experiment."

We shall now pursue our inquiry through other occupations and habits of life; and in proof that the severest muscular labour, continued through long periods of time, and under circumstances of the most trying character, is perfectly compatible with total abstinence from alcoholic liquors, we shall present our readers with a few selections from a large body of testimony which we have obtained from sources worthy of complete reliance.

A gentleman residing at Uxbridge thus writes:

"In the year 1841, I obtained the amount of bricks made in our neighbourhood by our largest maker; and the result in favour of the teetotallers was very satisfactory. Out of upwards of 23 millions of bricks made, the average per man made by the beer-drinkers in the season was 760,269; whilst the average for the teetotallers was 795,400—which is 35,131 in favour of the latter. The highest number made by a beer-drinker was 880,000; the highest number made by a teetotaller was 890,000: leaving 10,000 in favour of the teetotaller. The lowest number made by a beer-drinker was 659,000; the lowest number made by a teetotaller was 746,000: leaving 87,000 in favour of the teetotaller. Satisfactory as the account appears, I believe it would have been much more so, if the teetotallers could have obtained the whole gang of abstainers, as they were very frequently hindered by the drinking of some of the gang; and when the order is thus broken the work cannot go on."

Brick-making, we believe, is commonly accounted one of the most laborious of out-door employments; at any rate it is one which involves exposure to all the vicissitudes of weather, and therefore it may be taken as a fair sample of severe labour under trying circumstances. Respecting the above return, we think it should be especially remarked that it does not record the result of a trial made expressly for the purpose, with full advantages on both sides, and continued for a short time, during which the desire for victory might be supposed to lend an adventitious aid; but that it shows the actual amount of work done during an entire season by bodies of men working on both systems, but not pitted against each other.

The following statement by Mr. William Fairbairn, an eminent machine-maker, of Manchester, at the head of a firm employing between one and two thousand workmen, will be found in the Sanitary Report for 1840.

"I strictly prohibit on my works the use of beer or fermented liquors of any sort, or of tobacco. I enforce the prohibition of fermented liquors so strongly, that if I found any man transgressing the rule in that respect, I would instantly discharge him without allowing him time to put on his coat. In those foundries in which there is drinking throughout the works all day long, it is observed of the men employed as workmen that they do not work so well; their perceptions are clouded, and they are stupefied and heavy. I have provided water for the use of the men in every department of the works. In summer-time the men engaged in the strongest work, such as the strikers to the heavy forges, drink water very copiously. In general the men who drink water are really more active and do more work, and are more healthy, than the workmen who drink fermented liquors.

"I observed on a late journey to Constantinople, that the boatmen or rowers to the caïques, who are perhaps the first rowers in the world, drink nothing but water; and they drink that profusely during the hot months of the summer. The boatmen and water-carriers of Constantinople are decidedly, in my opinion, the finest men in Europe as regards their physical development, and they are all water-drinkers; they may take a little sherbet, but in other respects are what we should call in this country teetotallers." (p. 252.)

The following is the published testimony of Mr. Josiah Hunt, a well-known agriculturist in Gloucestershire, as to the efficient performance of harvest-work on the abstinence system. His experiment is further valuable as showing the positive advantage gained by the substitution of articles of solid food for alcoholic liquors of equal cost—a point of great economic importance to the labouring classes. After mentioning the terms on which his work had been done in former years (namely 8*s*. 6*d*. per acre, and an allowance of three gallons of cider, or an additional payment of 3*s*. per acre), he continues:

"I let 80 acres of grass to mow, harvest, and stack, to four of those who did the like last summer, with three others, at 8*s*. 6*d*. an acre in money; and instead of 3*s*. an acre for drink, an equal sum to be expended in the purchase of unintoxicating drink and food; on condition that neither of them should taste any fermented liquor during the progress of the work. Three of the men had signed the pledge in the previous winter; the other four did so about a fortnight after they began to work.

"They commenced on the 10th of June, and finished on the 26th of the next month; which was longer by two weeks than they would have been if the weather had proved fine. The whole of the work, without the least exception, was performed more to my satisfaction than ever was the case before. During the progress of it, they gave abundant proof that they were equal to as much work as any seven men in the neighbourhood; and also to as much as they themselves had been equal to at any time whilst taking intoxicating drinks. They were not picked men; four of them, about the respective ages of 55, 41, 30, and 29, having worked for me for several years; the others, aged 41, 30, and 20, having been engaged at various times in the spring, without any intention of retaining them during the summer; and that they were not of more than average strength may be inferred from the fact that I was told before they began,—'We know very well how your experiment will end; for there are but two men out of the seven that can do a day's work; they will be knocked up before they have mowed two hours.' At the end of the first day's mowing it was, however, found that they had done more than any other men in the neighbourhood; and as they thus proceeded without being 'knocked up,' the tables were turned, and I was then told that they performed so well in consequence of their good living. How this was obtained, I propose presently to show; but before doing so, I must, in justice to the men, add, that their conduct during the summer has presented a striking contrast to much that I have witnessed in ale and cider-drinkers. I have not heard any improper expression escape either of them during the whole period, and their general behaviour has been very creditable.

"Instead of intoxicating drink, they used tea and cocoa, sweetened with sugar or treacle, and skim-milk. The following are the quantities used, with the cost, viz.: 2lbs. of tea, 22lbs. of cocoa, 31½lbs. of sugar, 4½lbs. of treacle, and 60 gallons of skim-milk; all of which cost £3 12*s*., instead of (as at the rate of the cider last year) £12. There thus remained £8 8*s*. to expend in food; and for one shilling more than this sum, or £8 9*s*., they were enabled to procure the following, viz.: one hundred-weight of beef, one hundred-weight of bacon, four sacks of potatoes, and one sack of flour, with twenty pounds of suet for puddings; all of which 'good living,' be it remembered, was obtained out of the saving effected by the substitution of an unintoxicating drink for the intoxicating and expensive one of the previous summer." (Bristol Temperance Herald, Sept. 1841.)

As this, being the testimony of a single individual, might be thought open to question, we shall add a summary of the testimony of thirteen farmers and labourers in the neighbourhood of Bodmin, Cornwall, who have for some years been in the habit of prosecuting their harvest operations without any allowance of alcoholic liquors to the men engaged in them;—an equivalent of some other kind being of course given. The total number of acres of hay and corn harvested by them on this plan in 1846 was 1518; and if to this be added the quantity harvested by teetotallers who were mixed up with beer-drinkers, the total amount harvested on the abstinence principle by the farmers attending Bodmin market would not be short of 3000 acres. This, we think it will be allowed, is a scale of operations quite sufficient to afford satisfactory results. The testimony of those who employ none but total abstinence labourers is unanimous in favour of the system. "I feel assured," says one, "that work can be performed better on the teetotal principle; and that quite as much work can be done as on the use of alcoholic drinks. I am quite satisfied of total abstinence being more congenial to health, strength, and happiness in the harvest-field than the old drinking system, and am resolved as long as I remain a farmer to save all my hay and corn on that principle." "I am glad to inform you," says another, "that I have done my labour this harvest with comfort and contentment on the teetotal principle, as I have for the past *eight successive* harvests." "Our parish," writes a labourer, "is divided into small farms; and many of the farmers have their harvest-work done by men who have to go to the mine or stream-work, and do their day's work first, and then in the afternoon they go in the harvest-field; and most of them say they would rather have teetotal beverages than intoxicating drinks. I have passed through *nine* harvests on the teetotal principle, and three I have been on the cold-water system, and I find this is best of all. I have been at it for a month together, and I could always do my work to the satisfaction of my employers; and the men I have worked with have said that teetotalism is best." Another farmer says, "We have saved our hay and corn for eight or nine years to my satisfaction; the last two years the best of all. The last harvest has been passed with pleasure to the men (though not pledged teetotallers) and to myself. Not an oath nor an angry word has been heard. We have worked in times of necessity till 10 o'clock; and I never heard one say that he was tired. One of them cut an acre and a half of wheat after 2 o'clock; two acres a day per man was the average quantity cut; and they worked with such comfort to themselves, that they wish to go through another harvest on the same principle." "I have sent," writes another farmer, "the return of the hay and corn I have cut and saved this year; and I can say that I have done it much more comfortably on the teetotal principle than we ever did when we used malt liquor; the work-people have done their work well and with great spirit." "Without any brawl or anything uncomfortable," is the additional testimony of another. "As to the comfort of the plan," writes another, "I can say, the more I have of it, the better I like it. Never did I do my work so easily, nor enjoy my health so well as

I have since I abstained from all intoxicating drinks; and as to the work-people in the harvest-fields, all appeared to be pleased and satisfied; and some of them who were not teetotallers said, that they would sooner work on the teetotal plan than on the drinking system, if they could be attended to properly. My full conviction is, that if farmers would but put half the expense in solids and teetotal drinks for their men, that they put in beer and cider, their men would be better pleased, and their work be better done, and a great deal of sin would be prevented." Where an improved diet has been substituted, in the Bodmin district, for alcoholic drinks, it has been found that the labourers, like Mr. Hunt's, *increased* in weight during the severe labour of harvest, as much as 5lbs. per man on the average.

We think that we have now adduced sufficient testimony of the inutility, to say the least, of fermented liquors, as regards the maintenance of muscular strength in field labour. It is obvious that practice here fully bears out theory; and that the substitution of solid aliment containing the materials of muscular tissue, for a liquid which contains but little of these, and whose principal constituent is a heat-producing substance—never less wanted than when laborious exertion is being made under the summer sun—is attended with the very result which the physiologist would predict, namely, an increase in the amount of muscular substance, and consequently in muscular vigour. If we only go the length of admitting that they are unnecessary, the duty of doing our utmost to check their employment seems to us imperative; since it is the universal testimony of those who have fairly tried the abstinence system, that the temper and habits of workmen, who were previously "moderate drinkers," are in every way improved by it,—to say nothing of the avoidance of absolute intoxication with all its evils, which, although the most obvious, is not perhaps the most important result of the abstinence system—since for one *drunkard* there are scores who are injuring their bodies and souls, their families and their employers, and who are consequently in the end more or less burdensome to the public at large, by what is accounted amongst them but a *moderate* use of fermented liquors.

We shall next adduce evidence of the equal inability of alcoholic drinks to sustain the bodily powers in prolonged labour of other kinds; and we may first mention a very striking case which came within our own knowledge a few years since. A gentleman with whom we were then intimate, and who, though moderate in his own habits, was by no means a disciple of the total abstinence system, informed us that he had once had the command of a merchant vessel from New South Wales to England, which had sprung so bad a leak soon after passing the Cape of Good Hope, as to require the continued labour, not merely of the crew but of the officers and passengers, to keep her afloat by the use of the pumps during the remainder of her voyage, a period of nearly three months. At first, the men were greatly fatigued at the termination of their "spell" at the pumps; and after drinking their allowance of grog would "turn in" without taking a proper supply of nourishment. The consequence was, that their vigour was decidedly diminishing, and their feeling of fatigue of course increasing; as our physiological knowledge would lead us to expect. By our friend's direction, coffee and cocoa were substituted for the grog; a hot "mess" of these beverages being provided, with the biscuit and meat, at the conclusion of every watch. The consequence was, that the men felt inclined for a good meal off the latter, their vigour returned, their fatigue diminished, and after twelve weeks of incessant and severe labour (with no interval longer than four hours) the ship was brought into port with all on board of her in as good condition as they had ever been in their lives.—When visiting Messrs. Boulton and Watt's celebrated factory at the Soho, Birmingham, some years since, we were much struck by the Herculean aspect of a particular workman, who was engaged in forging the steel dies (used in coining) into the massive blocks of iron in which they are imbedded. This, we were informed, was the most laborious occupation in the whole factory, requiring a most powerful arm to wield the heavy hammer whose blows were necessary to ensure the union of the two metals; and involving also constant exposure to a very high temperature. The day was sultry and oppressive; and the additional heat of the forge was, to our feelings, almost unbearable. But we stood awhile, watching this gigantic workman, the girth of whose chest seemed twice that of any ordinary subject, whilst, naked to the waistband, and with the perspiration streaming down his head and body, he dealt the rapid and skilful blows of his ponderous hammer upon the heated mass. At the first pause, we asked him (from mere curiosity, for teetotallism was then scarcely talked of) what liquor he drank; and he replied by pointing to a whole row of *ginger-beer* bottles behind him, the contents of one of which he imbibed every ten or fifteen minutes. He stated, upon further questioning, that he found it quite impossible to drink alcoholic liquors whilst at his work; their effect being to diminish his strength to such a degree as to render him unfit for it.

This case might be regarded as a solitary exception; but the fact is, we believe, borne out by general experience,—men who have to carry on laborious occupations at a high temperature, as in iron-foundries, gas-works, sugar-houses, &c., finding that the use of alcoholic liquors, whilst they are so employed, is decidedly prejudicial to them. Most such men, however, are in the habit of drinking a moderate amount of beer or other fermented liquors in the intervals of their work, and many more drink to excess; the idea that such liquors enable them to support their exertion being a very prevalent one among all classes. The matter was long ago put to the test, however, by Dr. Beddoes, who, under a conviction of the worse than useless character of fermented liquors for this purpose, went to the anchor-forge in Portsmouth dockyard, and selecting a dozen of the smiths, proposed to them that six of them should drink only water for one week, whilst the others took the usual allowance of beer. The men, convinced that such a system would not answer, refused to try the experiment, and were only induced to do so on the promise of a reward if they succeeded in beating the beer-drinkers. On the first day the two sets of men were very much alike; on the second, the water-drinkers complained less of fatigue than the others; the third day, the advantage was decidedly in favour of the abstainers; the fourth and fifth days it became still more so; and on the Saturday night, the water-drinkers declared that they never felt so fresh in their lives as they had felt during that week. This result may fairly be viewed with suspicion, on account of the strong inducement which this benevolent but not always judicious physician had placed before the water-drinkers to procure their trial of his system; and it might also be objected that a week's experience was not enough to test it. There is ample evidence at the present time, however, contained in the various publications devoted to the Total Abstinence cause, that labour of the severest kind, and under exposure to the greatest vicissitudes of heat and cold, may be fully as well sustained without alcoholic drinks as with the most moderate and regulated employment of them. We shall not quote from these publications, however, because their statements may appear to bear the stamp of partiality. It is comparatively easy, it may be objected, to get up a body of evidence in favour of *any* system of quackery; but the *whole* truth must be known, before we can give assent to doctrines so completely opposed to the experience and common sense of mankind. We have already grappled with the latter part of the objection, and have shown that the experience of mankind at large is decidedly in favour of habitual abstinence from fermented liquors; and in regard to the particular question of evidence, we trust that our readers will give us some credit for discrimination when we state that we have ourselves collected and carefully examined a great variety of evidence from all parts of the kingdom, some of it furnished by unwilling and much more by indifferent witnesses. Among the documents which we have before us is a letter from a "moulder" in the Gorball's iron-foundry at Glasgow, containing the following statement: "I can assure you that temperance men can do more work and better work than those who use or indulge in spirituous liquors of any kind. I have joined the Total Abstinence Society *eleven years* ago; and from that day to this hour I have abandoned the use of spirituous drinks; and the happy result has been that I am better in health and abler for work than when I was indulging in the use of those delusive liquors." From Rotherham we have the testimony of 100 reformed drunkards, of various occupations; among them that of S. S., who has been a teetotaller now about seven years, and whose work is moulding iron plates for spades and shovels, which is, taking it throughout the day, one of the hottest and most laborious occupations known. We have received from Leeds the testimony of thirty-four men (and we are assured that many more might have been easily obtained), whose signatures are appended to the following statement: "We the undersigned, having practised the principles of total abstinence from all intoxicating liquors, for the several periods stated below, and having during that time been engaged at very laborious occupations, voluntarily testify that we are able to perform our toil with greater ease and satisfaction to ourselves (and we believe more to the satisfaction of our employers also) than when we drunk moderately of these liquors; our general health and circumstances have also been considerably improved." Of these men, twelve belonged to the class whose occupations are commonly regarded as peculiarly trying; seven of them being furnace-men at foundries and gas-works, two of them sawyers, one a whitesmith, one a glass-blower, and the last a railway night-guard. The duration of the periods of abstinence of these men ranged from one to ten years. The following is the experience of a wood-sawyer of Glasgow, whose very well-written letter now lies before us: "I have wrought at this laborious employment for twenty-six years in the city of Glasgow, fifteen years of which I was under the fatal delusion that these liquors were strengthening, and that my hard work required that I should use them for the purpose. I joined the Total Abstinence Society eleven years ago, and from that day to this hour I have abandoned the use of these drinks; and the happy result has been, that I have been enabled to endure more fatigue, do my work better, and do more of it, than when I was indulging in the use of these delusive liquors." The following is another very striking testimony, given by a nail-maker at Glasgow: "I have been a teetotaller these five years; and though I previously believed that strong drink was necessary to aid me in my work, yet since I have become an abstainer, I find hard work easier, and long hours more readily to be endured. I am also one of the Glasgow Fire Brigade, and was once at a great fire at Mr. Thompson's mill for *seventy-three* hours in succession, with nothing but coffee and ginger-beer, and endured while all my comrades were beat and fell away." In the month of April of the present year, the Temperance Society of Leeds closed their monthly meetings for the season with a "working-man's demonstration," at which the representatives of twenty-one laborious occupations publicly testified to the compatibility of hard labour with perfect health on the Total Abstinence plan. The duration of their trial was in no instance less than three years, and in many instances extended to eleven; the shortest of these periods being, we should think, quite sufficient to test the value of the system.

We do not think it necessary to adduce any further evidence in support of our main position, that total abstinence from fermented liquors is consistent with the maintenance of the most perfect health, even under the constant demands created by labour of the severest kind, or by extremes of temperature; and that, on the whole, the abstinent system is preferable, on physical grounds alone, to the most moderate habitual use of them. The most powerful claim, however, which the Total Abstinence advocates have upon public attention, lies rather (to our apprehension at least) in the moral benefits which their system is calculated to produce; and it is with reference to these that we would earnestly recommend our readers to examine for themselves, whether a great deal that is commonly believed as to the *therapeutic* use of alcoholic liquors is not equally baseless with the notion of the necessity of their habitual use for the sustenance of the body in health. There can be no reasonable doubt that a great deal more wine &c. is employed as medicine than there is the least occasion for. It is so pleasant a remedy, that we have recourse to it on the slightest occasion. People prescribe it for themselves, because they think they understand its action sufficiently well to supersede the necessity of proper medical advice, and because it is so palatable and comforting a draught. Other medicines are usually nauseous to the taste, and our patients are glad enough to get rid of them when they have done their work; but this is too frequently continued long after the purpose which it is supposed to answer is no longer required. And there is abundance of melancholy proof, that a craving for fermented liquors, which has ultimately led to habits of the most degrading intemperance, has been not unfrequently created, even in most delicate, refined, and high-principled women, by the habitual use of them when introduced under the guise of medicine by the physician. The records of Total Abstinence Societies, moreover, show that in a very large number of cases in which drunkards supposed to be reformed have "broken out" or returned to their intemperate habits, the cause of the relapse has been the use of fermented liquors under medical direction, the mere taste of which has excited the craving that seemed long subdued. Hence in some of the forms of "pledge" the promise is made to refrain from even the medicinal use of alcoholic liquors; which we regard as a most dangerous and unwarrantable proceeding, since there are cases (as we shall presently attempt to show) in which no other agents can have the same beneficial effect, and the difference may even be one of life or death. The proper course we apprehend to be, that those who take the total abstinence pledge should promise not to take alcoholic liquors, except when these are ordered by a qualified medical practitioner; and it is the obvious duty of the medical profession to refrain from ordering them, except where the indication of benefit to be derived from their use is of the plainest possible kind.

We believe that if the question of the therapeutic use of fermented liquors be placed in the same aspect as that on which we have on former occasions attempted to show that the action of almost all our remedies must be at present viewed,—namely, as quite open to that new kind of investigation which consists in the comparison, not of different methods of treatment one with another, but of the results of each method of treatment with the natural course of the disease,—a great deal of evil of various kinds will soon be done away with. At present, nothing in the annals of quackery can be more truly *empirical* than the mode in which fermented liquors are directed or permitted to be taken by a large proportion of medical practitioners. If their physiological action be really as grossly misunderstood as we deem it to be,—if their benefit can be looked for in little else than their stimulating effects, and the belief in their permanently-supporting character be really ill-founded,—if we are to distrust the grateful sensations which commonly follow immediately upon their use, and to look for evil in their more remote consequences (as the experience of the results of their habitual employment would lead us to do), —then it is obvious that a great change will be needed in our usual practice in this respect, in order to bring it into conformity with the mere corporeal requirements of our patients, to say nothing of its bearing upon their moral welfare. We shall not presume to attempt a full exposition of all the circumstances in which the therapeutic use of fermented liquors is indicated; but we shall endeavour to lay down a few general principles, based upon the data which we may derive from the phenomena of their physiological action, and from practical experience as to their habitual or occasional use in the state of health.

In the first place, then, we may lay it down as a general principle, that as alcohol cannot serve as a *pabulum* for the healthy tissues of the body, so it cannot give any *direct* support to the system in furnishing the materials of those morbid products, which frequently constitute a drain upon the system that may become most serious from its amount and continuance. But it will be said that ample experience has shown that the administration of fermented liquors, in cases of excessive purulent discharge (for example), is the only means of sustaining the feeble powers of the system; and we are not disposed to deny that benefit is derivable from them. But we believe that this benefit is to be looked for in their stimulating action upon the digestive apparatus, which enables it to prepare and introduce into the system such an amount of the nutriment that constitutes its real pabulum, as it would not otherwise be able to assimilate. We believe it will be found that if our *chief* trust be placed in fermented liquors in such cases, failure is almost inevitable; and that the *power* of the system will depend, not upon the quantity of wine or porter that can be poured in without intoxicating effects, but upon the amount of solid nutriment which the patient can digest by their assistance. The quantity of alcohol given should therefore be carefully regulated by this indication; and it should be reduced in proportion as the demand for nutriment is lessened, and the tone of the stomach improves. There is another large class of cases with which practitioners in large towns are especially familiar, in which it is of the utmost importance to sustain the powers of the system for a time against some depressing influence, even though there be no considerable demand for material in the form of an extensive suppuration or the like. Such cases present themselves especially in ill-fed and intemperate subjects, especially among such as have been exposed to the additional depressing influences of bad ventilation and drainage. Almost every disorder in their frames has a tendency to assume the asthenic form; and it is of the greatest consequence, as in the instance already alluded to, to obtain the assimilation of nutritious matter. Here too, we believe that fermented liquors are indicated, not so much as general stimulants, but as exercising upon the digestive apparatus an influence which no other remedy with which we are acquainted can so forcibly exert. But for this purpose we apprehend that the quantity requisite is far smaller than that which is usually administered; and that great injury is often done by over-stimulating the stomach, and thereby positively weakening its power of supplying the real wants of the system.

It is, again, by their temporary stimulus to the digestive operations, that fermented liquors seem to be *occasionally* useful during pregnancy and lactation. We believe that in every case in which the appetite is good, and the general system healthy, the habitual use of these stimulants is positively injurious; and the regular administration of alcohol with the professed object of sustaining the strength under the demand occasioned by the copious flow of milk, is one of the grossest pieces of quackery that can be perpetrated by any practitioner, legal or illegal. For alcohol affords no single element of the secretion; and if the materials of the latter are introduced into the system as fast as they are drawn out of it, there is no exhaustion. In a healthy subject, and under a proper system of general management, this will be the case; and alcohol can do nothing but harm. But there are cases,—very few, however, in comparison with the whole,—in which the conditions of pregnancy and lactation produce an irritable state of the stomach, that prevents it from digesting or even receiving that food which the system really demands; and in some of these we have known the regular administration of a small quantity of alcoholic liquor more efficacious than any other remedy. In one instance of this kind that fell particularly under our notice, in which the mother was most anxious to avoid the assistance of fermented liquors, the lactation must have been early stopped, on account of the want of functional power in the stomach, and the very poor quality of the milk, had it not been that the administration of a single glass of wine or tumbler of porter per day was found to promote the digestive power to the requisite degree, and thus to produce a general invigoration of the system, which was speedily manifested in the improved condition of the child as well as of the mother.

The small allowance we have mentioned never required an increase, and was relinquished without difficulty soon after the weaning of the infant.

We believe, then, that cases are of no infrequent occurrence in which, under some *temporary* depressing influence, the powers of the digestive apparatus are not adequate to supply the demand upon it made by the system, and that recourse may in such cases be advantageously had to alcohol as an equally *temporary* stimulus. But it is worthy of consideration whether, when it is thus administered for purely medicinal purposes, it may not be desirable to give it in such a medicinal *form* as will render it not peculiarly palatable or inviting, in order that the patient may have no inducement to continue the use of it after the real demand has ceased to exist.

There is another class of cases in which it appears to us that alcohol may serve a most important purpose that no other substance can answer. We refer to those in which there is a positive deficiency of heat-producing materials in the system, and in which the digestive apparatus is for the time incapable of introducing such as are ordinarily most serviceable for this purpose. Such a condition is the result of many exhausting diseases, and more particularly of certain forms of fever, in which, without any particular local affection, the powers of the whole system are prostrated by the action of a poison introduced into the blood. Day after day the fatty matter of the body is used up in the respiratory process, and no food is taken in to replace it; and thus, as in cases of simple starvation, the patients die of *cold* unless some means be taken to sustain their heat. Now there is reason to believe that when alcoholic liquors are received into the stomach they are taken into the circulation, not by the lacteals, but by the more direct channel afforded by the permeable walls of the capillaries of the mucous membrane. Theory would teach us that through such a thin septum the alcoholic fluid, being thinner than the blood, would pass towards the latter by endosmose; and experiment fully confirms this view, since it was found by Sir B. Brodie, that alcohol in strong doses exerts its usual effects upon the system, even though the thoracic duct be tied; and MM. Bouchardat and Sandras have obtained evidence of its presence in the blood of the gastric veins. Thus, then, alcoholic fluids introduced into the stomach can be directly absorbed, without any of that preparation which the oleaginous or farinaceous materials of combustion require; and we can well understand, therefore, how, in the advanced stages of fever, when everything depends upon the power of sustaining life until the poison has been expelled from the system, alcohol should be a more powerful therapeutic agent than any other. A severe epidemic of the kind we allude to (the *synochus* of Cullen), which we witnessed some years ago, afforded us the opportunity of seeing the results of opposite modes of treatment in two sets of cases as nearly similar as might be; in neither were any very decided measures adopted during the early stages of the fever, for none seemed called for; but in one set the same expectant practice was continued to the end, whilst in the other the administration of wine and spirit was commenced as soon as the weakness of the pulse and the coldness of the extremities indicated the incipient failure of the circulating and calorifying powers. The quantity was increased as the necessities of the patient seemed to require; and we remember one case in which a bottle of sherry and twelve ounces of whiskey every twenty-four hours was the allowance for some days. The result was that the mortality on the former system was at least three times as great as on the latter; the patients dying from simple exhaustion and cold, and no local lesion being detectible on post-mortem examination.

Now, in cases where alcohol is thus beneficial, there is an absence of anything like *stimulating* effects. The pulse is usually *lowered* in frequency, instead of being accelerated, and the brain is brought back to more regular action, instead of being disturbed. That a very large quantity of alcohol can be thus given without producing a stimulant effect (and the same is probably true of alcohol taken during exposure to very severe cold) is probably due to the fact of its being burned off almost as fast as it is taken into the circulating system, so that it never accumulates to such an extent as to act injuriously on the brain. We are acquainted with no case in which the beneficial influence of a particular remedy, when administered with caution and discrimination, is more obvious; and we would strongly urge upon those who *intemperately* (as we think) advocate the Total Abstinence cause, and who deny that alcohol *can ever* exert any beneficial influence on the human body, to consider whether so clear a case is not here made out, as to show that one exception at any rate must be made to their assertions.

These are the principal classes of cases in which the regular use of alcoholic fluids seems to us to be indicated. Of those in which their administration as stimulants is urgently called for, in order to sustain the flagging circulation when the heart's action is enfeebled by some violent shock to the general system,—such as concussion of the brain, a blow on the epigastrium producing concussion of the solar plexus, extensive burns or lacerations of the surface, severe and sudden hemorrhage, and the like, —we need say but little. Nothing can be more absurd than to say that, because alcohol is a poison, it can never be beneficial, since the same applies to every one of our most potent remedies; and those who declare that they had rather die than swallow a drop of this poison (and we have been assured that this threat has been acted on), seem to us to be as wilfully throwing away their lives as the suicide who takes a dose of prussic acid or blows his brains out with a pistol. In many of the cases we have mentioned, none but alcoholic stimulants will have the desired effect; and if that effect be not produced, death is inevitable. Those who have watched, as we have, by the bedside of children in a state of collapse from a severe burn, and have had the satisfaction of finding themselves able to sustain the circulation and the warmth of the body by the frequent administration of a spoonful or two of cordial, but have experienced the subsequent mortification of finding that, when they had given place to another less attentive nurse, the little patients have sunk after a brief intermission of the constant support which they require, can fully enter into our appreciation of the value of this class of remedies. But in all cases of this kind, it is of the utmost importance not to carry the stimulating plan too far, or the subsequent reaction may give us a fearful retribution for our incaution. And this is of course to be peculiarly borne in mind in cases of concussion of the brain; since the reaction which results from the injury alone is in many cases so difficult to combat, without the addition of that which results from the injudicious use of stimulants. We are inclined to think that, in many cases of prostration, in which the cooling of the body is taking place rapidly, and offers an additional impediment to the restoration of the circulation, the freer application of external warmth, especially by means of the hot-air bath, is likely to prove a most useful adjunct to the stimulants exhibited internally.

The class of cases in which the habitual use of alcoholic stimulants is most commonly, and in our opinion most perniciously, recommended by practitioners of medicine, is that in which there is chronic disorder of the digestive apparatus, with its multiform consequences. This disorder, in at least nine cases out of ten, has its origin in inattention to the laws of health, as regards diet, regimen, exercise, physical or mental exertion, and the like; and we cannot reasonably look for its cure by the use of stimulants. For the action of these, in such states of the system, is precisely like the application of the whip or spur to the horse already tired, which produces a temporary improvement in his pace, and prompts him to get through his work the quicker, but which leaves him, when he has done it, more fatigued than if he had taken his own time. We do not in the least deny that by men who are undergoing the excessive "wear and tear" of incessant and anxious mental exertion, the work is accomplished with more feeling of ease at the time, and even with less immediately consequent fatigue, when alcoholic stimulants are moderately employed. And upon such a system we find men going on month after month, and even year after year, without any obvious injury. But the time almost inevitably comes, when the overtasked system gives way; and long and difficult is then the process of restoration from its disordered state, as every medical man well knows. Now we are confident that when the exertion of the nervous system is greater than can be borne without the assistance of alcohol, provided due attention be given to diet, fresh air, out-door exercise, and sleep, the excess produces a positive injury, which is sure to manifest itself at some time or other; the use of alcohol only warding it off for a time, and preventing it from being at once felt. It is in renovating the system after such a course of long-continued ill-treatment, that we regard the hydropathic treatment as peculiarly effectual. We may keep our patient in town at his usual occupations, practise all kinds of experiments upon his stomach, recommend fat bacon or lean chops, prescribe blue-pill and senna-draught, or quinine and calumbo, and ring the changes upon all the wines and malt-liquors which the cellar can furnish, in search of one that shall be free from directly injurious consequences; but we shall not effect a twentieth part of the benefit which our patient will derive from giving himself a complete holiday, betaking himself to some agreeable spot where there is sufficient to interest him, but nothing to excite; promoting a copious action of his skin by exercise, sweating, and free ablution; washing out his inside with occasional (but not excessive) draughts of cold water, and trusting to the natural call of appetite alone, in preference to artificial provocatives. Let those who decry hydropathy witness the results of this method, as we have done, in but a few cases, and they *must* come to the conclusion, unless blinded by prejudice or interest, that water is better than wine, and that a hearty miscellaneous meal swallowed, with a vigorous natural appetite, is more invigorating than the carefully selected and delicately-prepared viands to which the dyspeptic subject is compelled to restrict himself, and which he can only digest with the aid of a glass of sherry, or a tumbler of bitter ale.

The insensibility to the effects of various morbific causes, which the use of alcoholic stimulants induces, and the toleration of them which it thus permits, is one of the most fertile sources of subsequent disease. As in the cases just adverted to, if we are prevented from feeling the *immediate* consequences of our improper course, we flatter ourselves that we are uninfluenced by them, and we give to our wine, our spirits, or our beer the credit of the escape. But this is far from being the case. The enemy is only baffled, not dispersed; and although he lies concealed for a time, he only waits until his onslaught can be more effectually made. Bad air, insufficient and unwholesome food, impure water, foulness of the skin and garments, and similar departures from the strict laws of health, *must* exert their influence on the system, all the alcohol in the world notwithstanding; and it is one of the greatest benefits of abstinence that, by making these evils less endurable, it prompts the sufferer to seek a remedy. Let our readers refer to the account of the former condition of the great tailors' workshops in London, (Sanitary Report, 1842, pp. 99 et seq.), where the heat and closeness were such that on the coldest nights of winter large thick tallow candles melted and fell over with the heat, and fresh hands from the country fainted away; and where gin was taken at seven o'clock in the morning to get the strength up for the day's work, and repeated three or four times in the subsequent ten hours: and then look at the consequences upon the health of the men, whose average age is not above thirty-two years, owing to the large mortality from consumption; whilst, at fifty, they are considered as superannuated. We have here an example that speaks strongly for itself. And applying this result to other cases, we think it will be admitted that when the *tolerance* of such nuisances as exist in the dwellings and workshops of our labouring population depends upon the use of fermented liquors, it is nothing else than an unmixed evil.

We might have added much upon other topics connected with the therapeutic use of alcohol; but our limited space compels us to leave it with only one observation. The whole medical art is based upon experience; and the value of any remedy can only be fairly tested by the omission of it in some of the cases in which it has been reputed to be most successful. Nothing can be stronger than the reputation which alcoholic stimulants have acquired, as affording efficient aid in the maintenance of the bodily strength under circumstances calculated to exhaust it; and yet the most unimpeachable testimony has shown the fallacy of this opinion, and has put "universal experience" quite in the wrong. So it has sometimes happened that medical men have assured staunch teetotallers that they would die unless they admitted alcohol into their system as a medicine; but the patients, being obstinate, did neither, thus falsifying the prediction in a very unexpected measure, and proving that the experience of doctors is not more infallible than that of the public.

We should gladly, also, have discussed the question, whether the sudden and complete disuse, or the slow and gradual diminution, of the allowance of fermented liquors, in cases where intemperate habits are to be reformed, is the least injurious to the constitution. But this, too, we must dismiss with the brief observation that, considering the large number of habitual drunkards who have adopted the former course, the number of cases of delirium tremens that have occurred in consequence has been marvellously small. If the moral strength could be relied on to adopt the more gradual method, we should consider it as safer on the whole; but in those who have been subjected to the degrading influence of frequent intoxication, and have acquired that craving for liquor which must be regarded as constituting a diseased condition, every taste of the forbidden gratification occasions a fresh conflict with the better nature, to which it is most dangerous to subject it, and the shortest method is generally the safest.

We now commend this important subject to the best attention of our readers. We speak as unto wise men; and we ask for nothing but a candid and dispassionate hearing.

---

